# A new method for detection of microbursts via point observation methods and field measurement for validation study with Doppler weather radar

**DOI:** 10.1371/journal.pone.0317627

**Published:** 2025-03-07

**Authors:** Ekim KÜLÜM, Mustafa Serdar GENÇ, Ferhat KARAGÖZ

**Affiliations:** 1 Wind Engineering and Aerodynamics Research Laboratory, Department of Energy, Systems Engineering, Erciyes University, Kayseri, Turkey; 2 Department of Electronics and Automation, Kirsehir Ahi Evran University, Kirsehir, Turkey; 3 MSG Teknoloji Ltd. Şti., Erciyes Teknopark, Kayseri, Turkey; 4 Turkish Accreditation Agency (TURKAK), Ankara, Turkey; King Fahd University of Petroleum & Minerals, SAUDI ARABIA

## Abstract

Wind shear (WS) phenomena are critical in many applications, especially in aviation, wind energy and urban planning. Microburst (MB) detection is important for ensuring safety during aircraft landing/takeoff, eliminating imbalances caused by shear from wind turbines, and for static calculations in urban planning. In this study, microburst events were detected using meteorological data. A new algorithm was applied to Light Detection and Ranging (LIDAR) data and 3 different cup anemometer data were available for 1-min and 10-min measurement periods. First, MB condition parameters using power law and basic wind shear analysis based on the scope of international criteria were defined, then checked in the algorithm. All results are compared with each other on behalf of detected microburst count, day, minute, and period. Detected events were matched at 66% and 85%, respectively, 10-min, and 1-min intervals. Validation studies were carried out for the same location by analysing the reflection values, reflection image and velocity product of the Doppler Weather Radar (DWR) with classical methods. However, when the radar results compared with 1- and 10-minute data sets, it was shown that 80% and 75% of daily events matched. The algorithm provided good continuity across LIDAR, different cup anemometers, and the weather radar. Consequently, the new algorithm will provide a great economic advantage.

## Introduction

With the decreasing trend in the use of fossil fuels, the need for renewable energy sources has increased. It is an inevitable fact that the need for electricity will continue to increase. The first method that comes to mind to fulfil the need is renewable energy sources. The most developed renewable energy source is wind energy worldwide. Therefore, the number of operating wind turbines is quite a lot. One of the operational challenges of wind turbines is the strong and irregular wind profiles. The definition of WS is a sudden change of wind speed and/or direction during flow. Turbulence and WS have very close definitions. WS is a kind of turbulence. But all turbulences are not WS. MB is the most common of the events known as the WS phenomenon. In aviation terminology, turbulence is considered an irregular event that occurs during aircraft flight. The WS is an irregular air event that occurs up to 1600 feet above ground level. Another frequently encountered expression in aviation terminology is clear air turbulence (CAT). Simply, CAT means turbulence in cloudless weather. But there are minor differences between the aviation authorities of countries. WS negatively affects living conditions as natural disasters. The occurrence of wind shear events can vary in a narrow area. So, it becomes important micro siting of wind turbines. The WS phenomenon adversely affects the mechanical parts of the wind turbines; therefore, it increases the operation costs of turbines. Detection and prediction of WS is of vital importance. LIDAR is used in wind energy applications for wind shear detection. Wind turbines are deployed in locations where the wind is continuous within a certain value range due to their operating principles. It is common to see extreme wind conditions where wind turbines are installed. Sudden changes in the speed and/or direction of the wind flow are a negative situation for wind turbines, especially for the blades. It causes accidents and early completion of the economic life of turbines.

**Fig 1:**
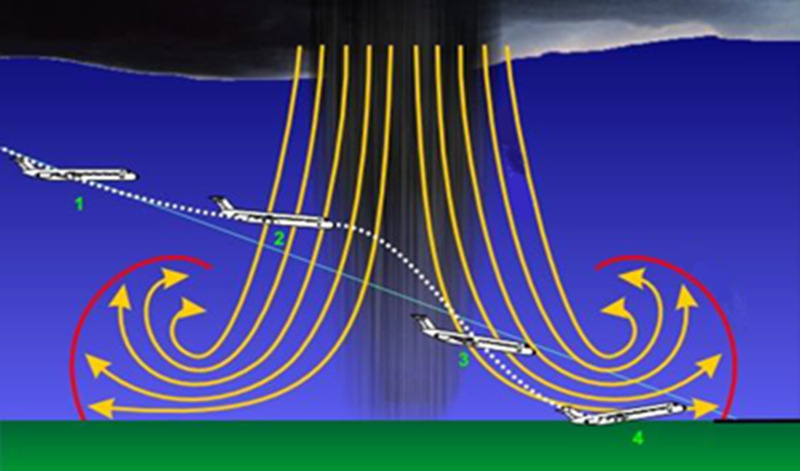
The schematic drawing of microbursts and possible aviation hazards of MB [9].

WS is also important in airport operations. The wind shear events during the landing and takeoff routes of aircraft pose a dangerous situation. In these situations, pilots can take measures such as passing the runway, increasing/decreasing the aircraft, going around the shear event, divert to another airport according to the International Civil Aviation Organization (ICAO) and the Federal Aviation Administration (FAA) definitions. More than 1500 people have died in aviation history because of WS incidents since 1943. MB is commonly seen among wind shear events (Fig 1). There have been 640 accidents caused by extreme wind shear events in the last 25 years [1]. WS research studies, independent of aviation, started in the 1970s. The Low Level Wind Shear Alert System (LLWAS) system was developed in 1983 under an agreement between the FAA and the National Center for Atmospheric Research (NCAR). Designed to provide information for each runway separately, the system was tested until 1988. In the current version of the system, which started with anemometers, many imaging systems such as radar, LIDAR, and satellite measurements can be included. Today, Phase 3-LLWAS systems are used with the algorithms developed [2]. Downburst (DB) and MB are the most seen events in the WS phenomenon. DB is more widespread and has a stronger impact than MB. The MB has a narrower area and less impact. Zhou et al. investigated the relationships between vertical wind shear, radiation, ice clouds and precipitation. In the analyses where wind shear was removed, it was determined that precipitation decreased [3]. Another study reported 927 downburst events, of which 914 were classified as microbursts and 27 as macrobursts. In this context, a prediction process was realized on cold pool strength, low-level lapse rates, WINDEX, lifted condensation level, DCAPE, WMAXSHEAR, derecho composite parameter, 2-m temperature, delta theta-e, and mean low-level relative humidity data [4].

Studies on MB detection in the literature are quite limited. The number of experimental studies is also rare. Zhang et al. created MB events using a jet engine. They found that rotary-blade wind turbines were more affected than fixed-blade wind turbines. Studies on the effects of MB events on wind turbines have been examined. In the experimental study, it was observed that when the MB event occurs, the average loads increase up to 4 times and the surge loads increase up to 10 times [5]. There is another important experimental study that has been conducted by L. Zhang et al. Sand and dust storms were investigated using Doppler and LIDAR. The WS index is examined by different methods and important information about sand and dust characterization is given [6]. The tallest meteorological tower data analysed is called Shenzhen Meteorological Gradient Tower and is 365 m high. Mean wind speed, momentum flux and drag coefficient investigation results can help avoid the hazardous effects of tropical cyclones in coastal regions. Turbulence Kinetic Energy (TKE), shear production, shear production rate and dissipation rate have been calculated and compared [7]. This will harm the economic life of wind turbines. In addition, it will increase maintenance costs. Nguyen et al. used a deterministic-stochastic hybrid model to predict the wind turbine loads of DB events [8]. One of the most associated points of MB is the cumulonimbus cloud. The MB event lasts between 2 and 5 minutes over an area of less than 4km.

**Fig 2:**
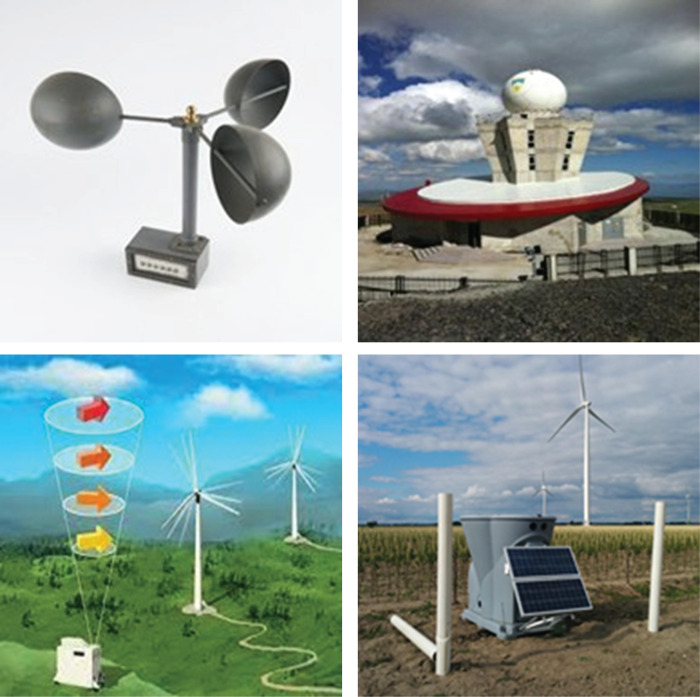
a) Anemometer [12] b) TDWR [13] c) LIDAR d) SODAR [14].

Different equipment can be used for wind shear detection [10]. Mainly, anemometer, Terminal Doppler Weather Radar (TDWR), satellite imaging, LIDAR and Sonic Detection and Ranging (SODAR). In practice, at least two of these devices are generally used [11]. An anemometer is an instrument for measuring the speed of air movements in horizontal or vertical directions (Fig 2a). It is the most common and reliable wind speed measurement method. Anemometer measurement is the most frequently used method in wind energy applications. It is used to determine the wind potential of areas where wind energy investment is considered. It is also used for performance tests of wind turbines.

One of the shear detection remote sensing devices is Doppler Weather RADAR (DWR) shown in Fig 2b. The signals sent through the antenna are reflected by clouds, water drops, snow, and topographic obstacles. The receiver receives and processes the reflected signals to generate data. The antenna transmits/receives signals at different angles for periods of a few minutes. Another wind shear detection device is LIDAR shown in Fig 2c. It is the calculation of meteorological parameters such as speed and direction at the height of the reflection by processing the frequency of the signals received from the reflection of the laser beams sent from the device. SODAR is the measurement of meteorological parameters such as wind speed and direction at various heights by processing the signals received as a result of the reflection of sound waves hitting the particles in the air (Fig 2d). SODAR is used mainly in wind energy applications. The devices mentioned above are widely used in WS detection. Except for the anemometer, the other devices are classified as remote sensing.

Kusuma et al. conducted a comprehensive literature review on wind shear detection. In their study, they argued that TDWR is more successful in WS detection than DWSR [15]. Hong Kong International Airport (HKIA) uses a WS detection system using LIDAR, TDWR, weather stations and buoys as wind shear detection systems. The MB algorithm was tested using numerical weather prediction (NWP). In HKIA, 3 different MB detection methods are used. Among the algorithms tested by estimating using NWP, the glide path scan wind-shear detection algorithm gave the best result [16]. On the other hand, MB detection is also important for the protection of habitats. MB events can also damage constructions and trees. Work is being done to route MB events and the MB’s progress [17]. Ibrahim et al. developed a new method using NEXRAD Radar data and ASOS data and compared the combination of Plan Position Indicator (PPI) scanning [18]. In another case study, satellite data, radar data and pluviometry station data were processed for the events on the Rimac River from February 15–18, 2018 [19]. A study on MB detection measured the accuracy of the PPP scheme [20]. Ratsanari et al. considered the Low-Level Wind Shear Alert System (LLWAS) system at Soekarno-Hatta International Airport. On April 5, 2018, they analysed the Doppler Radar and Automated Weather Observing System (AWOS) data. 15 flights were subjected to delays, overruns and diversions at the time of the incident during MB events [21]. Another study investigated the WV characteristics at Hong Kong International Airport using LIDAR data and machine learning techniques. A new algorithm was developed for Wake Vortex (WV) detection using Range/Height Indicator (RHI) outputs [22]. As mentioned before, there are many applications and academic studies in which WS detection is performed using different combinations of RADAR, LIDAR, SODAR and AWOS hardware. However, with the advancement of software technology, the use of machine learning, and neural networks are increasing [23]. Wang et al. investigated the wind resistance of buildings for complex fields. They developed a new machine learning method called CondNN. The results show new method is useful for similar applications [24]. Yuan et al. proposed a new WS detection scheme using only coherent Doppler LIDAR data. There is another interesting study that has been conducted by Liu et al. at Beijing Capital International Airport. They carried out a comprehensive study on the detection and effects of wind shear events caused by buildings. The data was collected by LIDAR and validated with sonic anemometers. The LIDAR design strategy was modified to determine the shear effects of buildings in and around the airport. Wind shear caused by buildings increased the headwind turbulence intensity and affected the wind speeds in the wake regions [25]. Yuan et al. handled an experimental study about downburst characteristics in wind tunnels. Downburst flow has occurred in the tunnel via a designed active-controlled multi-blade system [26].

In general, radar technology is the most advantageous device in this regard among the technologies mentioned. However, radar cost is also an important item in WS detection systems. Antenna studies in literature also make important contributions to WS detection. The development of antenna technology will improve both the accuracy of the devices and the cost savings [27]. With the development of antenna technologies, radar antennas will be able to decrease in size and increase in function. Therefore, the combination of radar technology and antenna technology will pave the way for new studies on WS detection, especially the rain effect [28]. There is another radar type is synthetic aperture radar (SAR). Although SAR is used to determine landforms at high resolution [29], it can also be useful for WS studies. Detection of natural structures such as cliffs is important for shear applications because these structures are vortex-forming factors.

In this paper, a new approach using a single-point measurement station is presented MB detection. Data includes anemometers, wind direction sensors, temperature humidity and pressure sensors. MB detection is performed using collected data from the stations. The method used provides a new method to the literature on MB detection. MB characteristics were realized with Python using wind speed, wind direction, temperature, humidity, and pressure data. Identified shear events have been shown on the designed interface. The data was collected in Aksaray, Turkey between Feb and Dec 2018. Then a validation study occurred. Karaman DWR data was investigated according to the MB phenomenon at the same time as the new algorithm data time. The new method and radar results are well-matched. On the other hand, a new wind shear method has been applied to the 1-min interval data. Results are better than the 10-min interval for the same algorithm. MB events were detected using DWR data and the developed algorithm was tested on the behalf of day, minute, and period of MB. Also, a time interval of measurements is compared with the MB detection. With 3 different measurement technologies, 4 different measurement points and 5 different data sets, this is the first time such a comprehensive comparison and validation study has been performed in the literature.

## Materials and methods

### Data collection systems and data handling

The data was collected by various methods. All data were provided in raw format. The data handling processes were applied. Table 1 shows the date range of considered data. 4 different data sets were processed under the new MB algorithm perspective. Radar data were evaluated with a standard method. Aksaray wind measurement station and LIDAR collected data near each other. In Aksaray station, wind collection was handled by a cup anemometer. LIDAR collected data through remote sensing technology via laser waves. Both data set time intervals were 10 minutes. This provided us the opportunity to compare systems operating with different measurement principles at the same location. Hasan Dagi station data was also collected with a cup anemometer. There were two data sets from this station, the first was 1-minute measurements and the second was 10-minute measurements. The evaluation of the average measurements taken from the same point with different periods allowed us to see the response of the algorithm in this regard. The DWR data was processed for 5 days. Because the Turkish Meteorological State Service shared the radar according to the legislation of “PROCEDURES AND PRINCIPLES REGARDING THE SCOPE AND NATURE OF METEOROLOGICAL DATA AND PRODUCTS”. Moreover, Hasan Dagi station data shared as same legislation for this study as mentioned period below [35].

**Table 1:** The time intervals of 3 different stations and 4 different data sets analysed with the developed algorithm. Compared with Karaman Radar data time range and with each other.

Data Name / Time Range	Jan (2018)	Feb	Mar	Apr	May	Jun	Jul	Aug	Sep	Oct	Nov	Dec	Jan (2019)	Feb
Karaman Doppler Radar														
Aksaray Wind Measurement Sation (10 min interval)														
Hasan Dagi Synoptic Station (10 min interval)														
Hasan Dagi Synoptic Station (1 min interval)														
LIDAR Measurement (10-min interval)														


$$\bar{\bar{v}}=\frac{1}{n}\overset{n}{\underset{i}{\sum }}v_{i}$$ Eq. 1

Developed software started with data handling processes for all point measurement sets. Icing, faulty, and empty data were removed from all data sets. Before WS studies on data sets, all data were compared with each other. Average wind speed data were calculated according to their interval between measurement times using Equation 1 [30]. According to the results, the mean wind speed in the Aksaray wind measurement mast is 6.48 m/s. The maximum turbulence intensity is 0.114 and the mean temperature is 12.91 °C. In Hasan Dagi synoptic station (10-min interval), the mean speed is 5.29 m/s. The difference in the mean wind speeds is 1.19 m/s, because of measurement height (101 m and 10 m). In Hasan Dagi (1-minute interval), the mean wind speed is 8.35 m/s. Additionally, the mean wind speed of LIDAR is 6.48 m/s at 101 m. The mean wind speeds are like each other at the same height measurements. Also, different measurement values are acceptable with higher mean wind speeds. These results have provided trustable clues at the beginning of the study.

**Fig 3:**
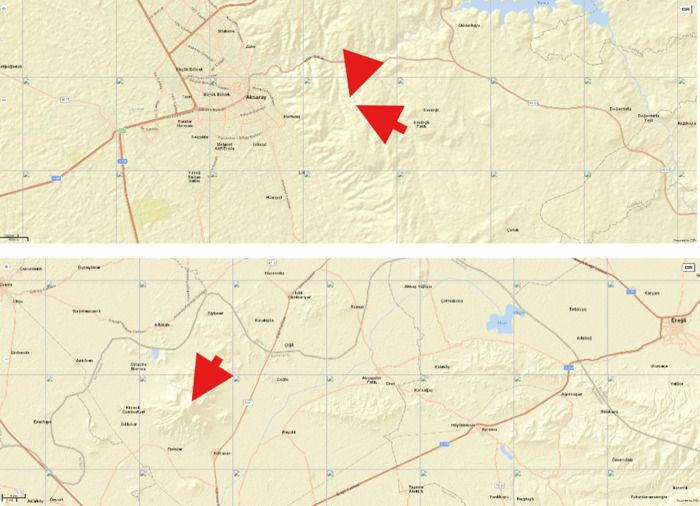
a) Location of LIDAR and wind mast measurement b) Location of RADAR.

One of the important parameters in WS detection is radar coverage. There are 3 measurement points within the area and 4 data sets have been compared to radar products. Fig 3 shows the location of LIDAR, wind mast and radar. All measurement points are in the radar product area and at low altitudes. On the other hand, Table 2 describes the technical specifications of point measurement devices (LIDAR, Anemometers). The accuracy of devices is especially critical for this paper.

**Table 2:** Technical information of measurement devices. Hasan Dagi station wind measurement data specifications taken from TSMS specifications and other equipment specifications have taken from the datasheets.

Parameter	Specification
Hasan Dagi Station (TSMS)	
Measurement range	0–65 m/s
Resolution	0.1 m/s
Accuracy	0.5 m/s
Threshold	0.5 m/s
Communication	Analog
Frequency	NA
Wind Measurement Station - Anemometer (Thies Clima4.3351.10.xxx) [31]	
Measurement range	0–75 m/s
Resolution	NA
Accuracy	0.2 m/s
Threshold	0.3 m/s
Communication	Digital
Frequency	1082 Hz
LIDAR (ZephIR 300) [**32**]	
Measurement range	1–70 m/s
Resolution	NA
Accuracy	0.1 m/s
Threshold	1 m/s
Communication	Digital
Frequency	50 Hz

*Accuracy and frequency are important for wind measurement applications. These values are close to each other in this study.

Radar data provided by Turkish State Meteorological Services. The closest radar images to the measurement locations belong to Karaman radar. The radar is C type Magnetron. Karaman radar was installed in 2015. The minimum antenna angle is 0.2^o^ and the maximum antenna angle is 45.0^o^.

### Detection of microburst by doppler weather radar

**Fig 4:**
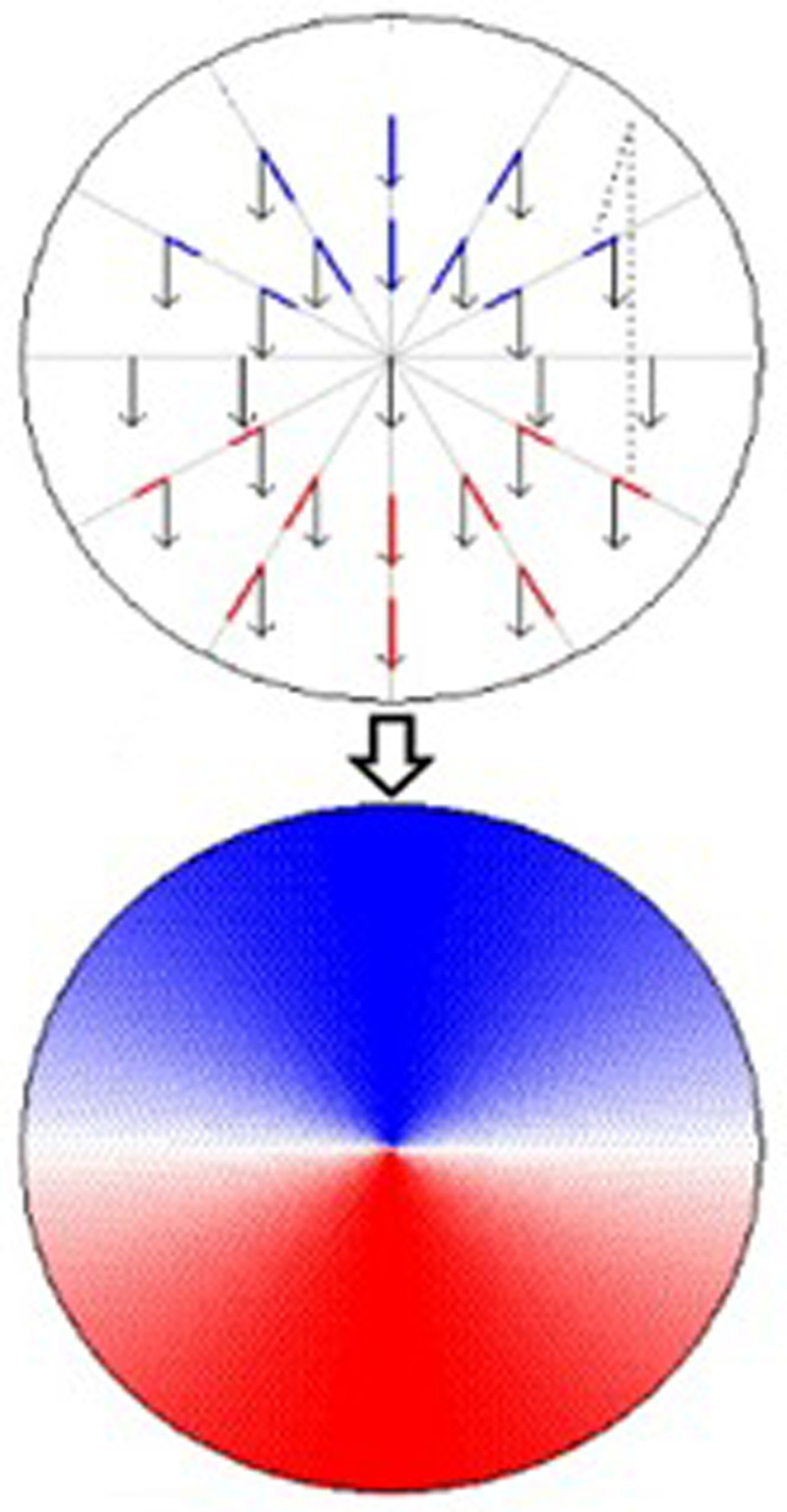
Image of the radar signal at a stable wind speed [38].

Doppler radars generate electromagnetic waves and transmit signals of a specific frequency to the target through an antenna. The wave is generated by Stabilized Local Oscillator (STALO) equipment. The purpose of weather radars is to target precipitation signals reflected from precipitation such as snow, rain and hail to reach the radar again. DWR can even detect liquids and frozen precipitation within rain clouds. The frequencies of the signals returning from moving and stationary targets are different from each other. If the target is stationary or moving at a constant distance from the antenna, the reflected frequency oscillates at the same frequency as the output frequency. If the target moves closer to the radar, the vibration frequency increases by V/λ. One of the main advantages of DWR is the ability to detect the movement of the target relative to itself. This makes it possible to measure the amount of precipitation and the radial speed of the wind. These products of radar are important for MB detection. Fig 4 shows the marking principle of radar. The radar is in the center according to Fig 4. The meaning of the blue markings indicates that the wind is moving towards to radar. Red marking is wind moving away from the radar [33]. Also, the investigation of wind rows in the products according to the radar center is a clue of the wind shear. The wind relative to the radar position is why the colors are different. Shear evaluation is made according to the deviations of the arrows shown in the picture.

Radar is a remote sensing technology that provides information about the speed, location, type and severity of meteorological events by scattering electromagnetic waves. Radar generates data by determining the direction and size of particles in the air. This data can be processed to detect and predict many meteorological events. These data are called products. Some of the products are;

Z =  Reflectivity (dBZ): It refers to the power of radar electromagnetic signals reflected from the target.

V = Velocity (mm/h): Indicates the speed of air masses.

W = Spectral Width (m/s): Spectral width indicates WS and turbulence.

ZDR=Differential Reflectivity (dBZ) Spectral width indicates WS and turbulence [27].


$$\text{ZDR =10 log10 }\left(\text{zh/ zv}\right)$$ Eq. 2

R = Precipitation(mm/h): Its spectral width indicates WS and turbulence [27].


$$\text{Z = A}{\text{.R}}^{\text{b}}$$ Eq. 3

A and b are experimental constants and are assumed as Z = 200 and b = 1.6 [34].

PPI and CMAX (Column Maximum) data within radar products are frequently used in WS detection. It is the display of echo signals received from the antenna in polar coordinates at the desired altitude and azimuth. PPI results include Z, V, W, ZDR and R products. MAX results include Z, V, W and R products. The PPI image is the product of the radar coverage area overlaid on a topographic map. The CAPPI image is used to locate extreme events by analysing the vertical cross-sections (RHI).

The MB events detected using the data measured at the meteorological station were compared with meteorological radar products and values. The dates of MB events detected by the developed algorithm were matched with the dates of MB events detected from meteorological radar images. Then, the onset dates of rainfall were extracted from the dBZ values taken as the reflection value of the radar and the onset times of the MB events detected by the algorithm were compared. The data used in this study was collected by Vaisala Doppler Radar located in Karaman/Turkey. The software of radar is called IRIS. Reflectivity values have been calculated as the range of dBZ is ‒31.5 to + 95 [35].


$$z=\frac{\rho_{r}2}{LCK}$$ Eq. 4

**Fig 5:**
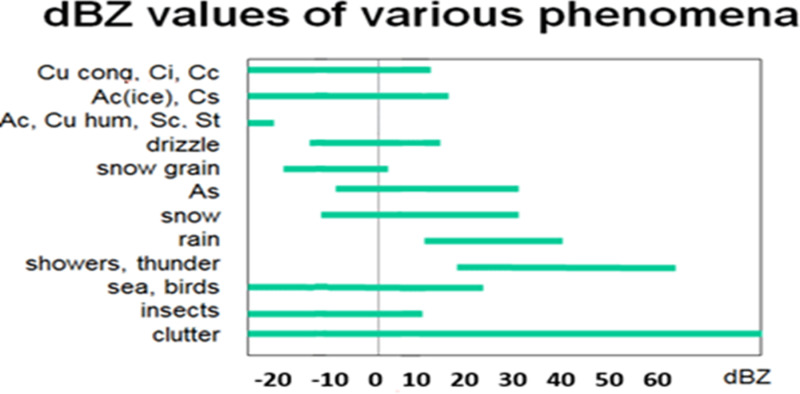
dBZ values of various meteorological events. [**36**].

P is measured as average power (Watt). r is the range to the bin. L is attenuation. C is radar constant. K is the refractive index and depends on the dielectric properties of the particle [35]. The meteorology of the process is the received signal power value multiplied by the square of the distance divided by the attenuation, hardware constant and precipitation constant. Fig 5 shows the dBZ values of some phenomena.

**Fig 6:**
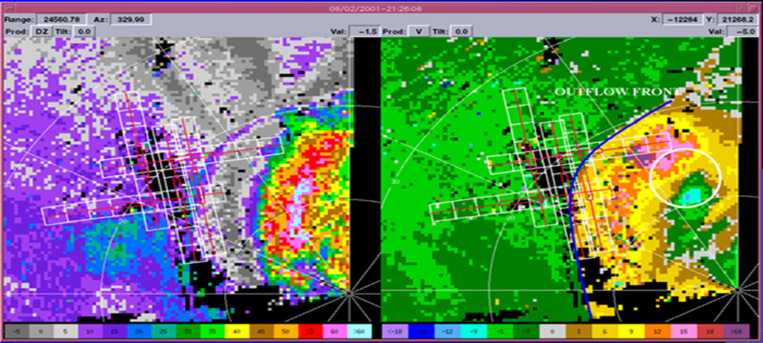
TWDR MB Image (White Circle) a) Reflectivity production of Radar b) Velocity production of Radar [37].

One of the important methods used in wind shear detection is the analysis of meteorological radar data. Parameters followed in this regard are convective clouds. WS is detected by tracking the location of convective clouds on the radar image. When the reflection products are analysed, linear moving clouds indicate the MB event. Fig 6 shows the MB event detected with TWDR [37]. When detecting the event, the antenna angle should be at minimum angles such as 0.1^0^ - 3^0^.

When detecting the MB event with DWR, 2 products are generally analysed. The first one is the reflection product. Its variability of up to 50dBz values within a 4 km area is one of the pieces of evidence of MB (Fig 6a). The other product is the radar velocity output. Due to the characteristics of MB, the microburst that occurs frequently changes in different directions in terms of velocity. In this case, the radar velocity product contains different colors together in a narrow area (towards and against the radar Fig 6b) [37]. When the conditions mentioned in both products are observed, it is great proof of the MB phenomenon. However, this applies to wet MB. Because airborne particles facilitate radar detection. Dry MB events are more difficult to detect.

MB incidents are categorized by FAA and ICAO authorities. According to the FAA, there should be a wind loss of 30 kt within less than 4 km. MB events have a maximum duration of 10 minutes. Approximately 50% of the last 5 minutes or less. MB generates high pressure and horizontal shear.

One of the most common methods to detect MB incidents is velocity images from meteorological radars. Due to its characteristics, MB occurs in a short time and the event ends in a short time. This makes detection difficult. In radar speed images, the presence of opposite speeds together in a small area is a clue to the occurrence of an MB event.

A realistic approach to wind shear detection with Doppler radar is the simple adjoint (SA) method. This method uses reflection and radial wind products from radar data [38]. Gu et al. modified this method and performed an experimental study. They performed MB and GF detection with the modified method [39].

### Detection of microburst by synoptic stations and wind measurement station

Turbulence intensity (TI) is an important parameter for wind shear evaluation. Turbulence is defined as sudden irregular air movements. Turbulence intensity is calculated by dividing the standard deviation by the average wind speed. TI is classified into three categories: low, medium and high. All measurements were analysed according to the TI classification. This step provides important insights into MB detection by examining the TI values before the MB detection algorithm. I, *σ*, *v* are defined as TI, standard deviation, and mean wind speed, respectively [40].


$$I=\frac{\sigma }{v}$$ Eq. 5

The data set specified in Table 1 was used in this study. An algorithm for MB detection was developed using Python software. In the data sets, the data handling process was performed first. Erroneous data due to freezing, malfunction and incorrect measurements were removed. The data handling step provides a more accurate for analysing results. Then, the comparison method, which is one of the basic indicators of irregular wind detection, is applied. The difference between the wind speeds measured with two different devices at the same height is evaluated. If this difference is greater than 0.5 m/s, it is a sign of WS. Alternatively, in case there are no measurements at the same height in the data set, the Log Law method is used to collapse the low-level wind measurements to the target level [40].


$$v=v_{0}ln\left(\frac{Z/Zs}{Z_{0}/Zs}\right)$$ Eq. 6

where v, v_0_, z, z_0_, z_s_ determine wind speed, measured wind speed, desired height, measurement height and surface roughness, respectively [40, 41].

Our second stage is power law control. The Power Law method is one of the leading methods used to calculate the reference wind speed in wind energy applications [11,40].


$$\alpha =\frac{ln\left(\nu ₂\right)-ln\left(\nu ₁\right)}{ln\left(z₂\right)-ln\left(z₁\right)}$$ Eq. 7

ν refers to wind speed at different heights. z refers to heights. The WS coefficient (WSC (α)) helps calculate wind speed for heights different from the reference height as shown in Eq 7. The α value is accepted as 0.143 in many applications. Experimentally, this number refers to natural wind conditions. The contribution of this method to the algorithm is that since the WS wind flow is known to be outside the normal conditions, values that differ from the standard α value are again accepted as WS evidence [40].

The next step is to specify the MB event within the algorithm. The wind speed, wind direction, temperature, and humidity values of the MB event are categorized as event-specific and the times when the MB event occurred in the data set are filtered.

**Fig 7:**
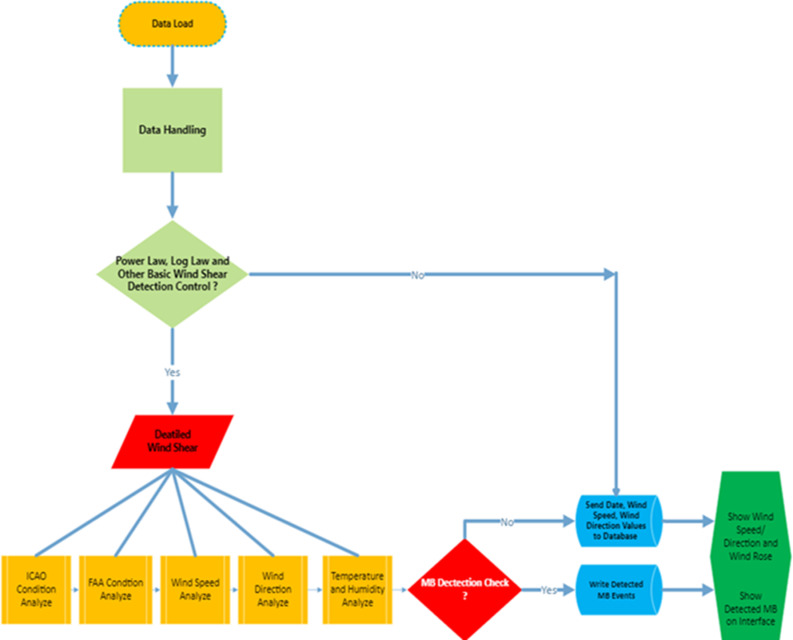
MB detection algorithm.

**Fig 8:**
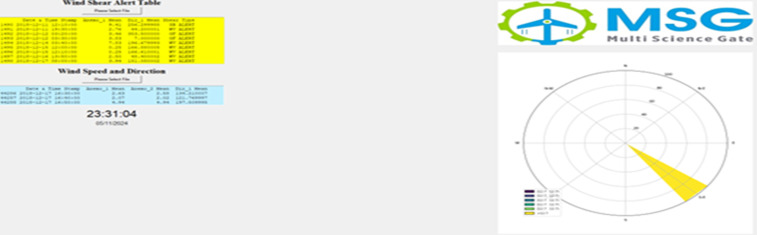
Wind Shear and Wind Speed/Direction guide.

The algorithm consists of interdependent conditions and sub-conditions. The first step after data loading is the data cleaning step, which is commonly performed in big data analysis applications. Then, the main clues of wind shear are analysed in the data set. The data resulting from the analysis, -ICAO, FAA, wind speed, wind direction and temperature/humidity-are examined and then the MB decision phase is passed as Fig 7. Only the algorithm was not created within the scope of the study. An interface and database were created to enable the algorithm to be displayed and to realize a user-friendly operation. The interface provides instantaneous wind speed and direction information and detected MB events with date, speed and direction information as Fig 8.

Before the “data loading” part, data were collected at the times specified in Table 1 and with the devices specified in Table 2. LIDAR and anemometer data were saved as.txt or.xlsx files in their own data logger systems. Data logging system files include mean wind speed, mean wind direction, mean temperature, mean humidity, mean pressure, battery status, collection time values and their min-max values. Only require time, average wind speed, average wind direction, average temperature, average humidity and average pressure information for the algorithm to both speed up the algorithm and prevent data clutter.

The MB detection algorithm starts with the data load step as shown in Fig 7. This means all collected data is imported to Spyder and PyCharm programs for the Python algorithm. Then one of the most important steps is applied which is called data handling. This stage is the part where frozen, corrupt and un-processable data is cleaned from the entire data set. As mentioned, power law and log law coefficient values are indicators of wind shear. So, these processes are applied before the detailed MB detection step. The algorithm goes through different processes in case the event is detected or not. The detected events are recorded in a different database and sent to the interface. In cases where no event is detected, instant meteorological information is displayed on the same interface. All systems are designed as usage of for end users. In other words, detected events are recorded and end-user control and algorithm accuracy are monitored. In the detailed wind shear analysis part of the algorithm, the MB characteristics determined by authorities such as FAA and ICAO are checked. These stages are also compared comprehensively with other MB meteorological features such as speed, direction, temperature and humidity.

The algorithm starts with the data handling part. When the temperature value cannot be detected from the data logger, the data section shows ‒99.99 in the file. After examining the historical climate characteristic of Aksaray, temperatures outside the range of ‒40 to + 50 were removed from the analysed data. Data sets during maintenance and repair within this temperature were also excluded from the analysis. The algorithm repeats these steps for other values within its limitations. It should be noted that it is not applied to cut-in and cut-off speeds for wind measurements. In the first part of the WS detection, a difference of 0.5 m/s between consecutive values. This condition is designed alternatively depending on the number of point measurements number. The WSC result calculated and controlled a 10% difference according to the neutral condition. If only one wind speed data set was available on site, the algorithm automatically generated a new wind speed data set under the log-law method for site-specific. After all of these processes, a detailed MB analysis is performed. ICAO and FAA wind speed loss parameters are controlled. Then wind direction should be between 80^0^ –100 ^0^ and 250^0^–270^0^. Different humidity and temperature values are controlled for wet and dry MB installations. The conditions described in the literature have been used to determine the measured MB durations.

## Results and discussion

Within the scope of the study, MB detection was performed by analysing the point measurement data with a newly developed method. To determine the accuracy of the improved algorithm, dBZ and velocity products of Karaman Doppler Weather radar obtained from the Turkish State Meteorological Service were used.

The wind speed, wind direction, temperature, humidity, and pressure values of the point data set was analysed holistically with the newly developed algorithm. The shear events detected by the algorithm are saved in a new Excel file. Then, with the interface created, instantaneous shear events and instantaneous wind speed and direction information are displayed to the end user (Fig 8). Various data sets are provided to us to evaluate the same point with different time interval comparisons in the same point measurement. Also, the data sets contain LIDAR and anemometer measurements at the same height and the same point. Algorithm responses of different devices evaluated. Validation studies have been conducted with radar products.

### Synoptic station results

There are 2 data sets provided by the Hasan Dag synoptic measurement station by the Turkish State Meteorological Service. The first one is collected in 10-min intervals and the other one 1-min interval. Differences in measurement frequency provided critical information about WS detection. Examining the detection of different measurement intervals of the same station against the algorithm has provided important information in terms of wind shear detection and comparison of detected events. The total measurement period for 10-minute interval data is 1 year, from 01.02.2018 to 31.01.2019. For 10-minute interval data, the total measurement period is 1 year, from 01.02.2018 to 31.01.2019. The last month of this measurement period covers 1-min of data which is between 01.01.2019 and 31.01.2019. Mean wind speed values are 5.29 m/s and 8.35 m/s respectively. Also, it is important to know that the wind data of the overlapping dates are compatible with each other increasing the confidence in the MB detection. It is critical for the 1-minute and 10-minute analysis of data on behalf of the same measurement period. Mean wind speed is 8.35m/s and 8.38m/s respectively for 1 min and 10 min. TI values are close to 0.126 and 0.124. Wind speed statistical analyses have occurred for all data sets and also for the same measurement period. However, other meteorological analyses occurred only for the same measurement periods. Mean temperatures are the same at ‒2.38 ° C. Average humidity is also 77.84% less than 0.02% from 10 min. There is not a huge difference between 1 min and 10 min statistical analysis. But there are more different results on behalf of MB.

The algorithm flow shown in Fig 7 was implemented in two datasets using Python software. On this scope, 51674 data rows in the 10-minute interval data set and 44661 data rows in the 1-minute data set was analysed. The results are impressively good. A total of MB events were detected in the data on 27 different days in a year, 12 of which were in January 2019. In the minute measurement, this value is 14 days. When MB incidents are counted annually, our newly developed algorithm detects 53 events. 24 of them have occurred in January 2019. The value is 33 in determinations made with minute measurements. It is important to note that in January, the days of MB events detected with the data with 10 minutes are 100% accurate with MB events detected with all minutes of data. The last comparison of 2 synoptic stations is that the detected MB phenomenon has been evaluated on behalf of characteristics of MB, duration of MB and count of detected MB. According to the result accuracy result is 79%. It should be known that these 2 stations distance between is approximately 24 km.

**Fig 9:**
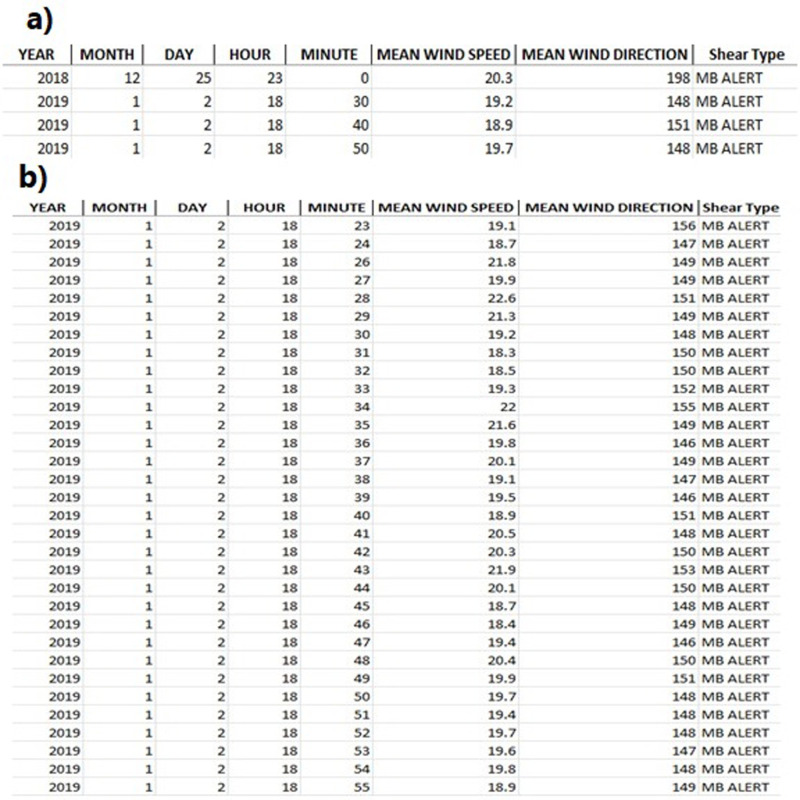
Microburst detection results of Synoptic Stations. a) Hasan Dagi Synoptic Station 10-min interval b) Hasan Dagi Synoptic Station 1-min interval.

**Fig 10:**
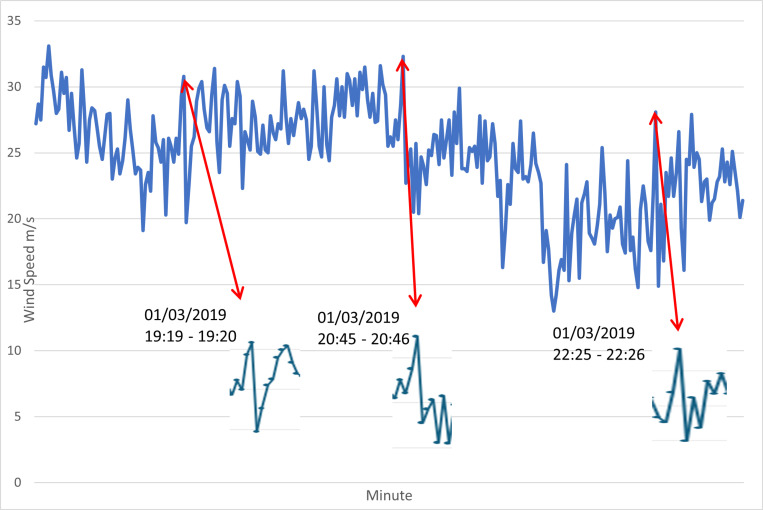
The areas indicated on the graph show large changes in wind speed within a minute. this is one of the important types of evidence of wind shear [42]. The highlighted periods overlap with the periods detected by the algorithm.

Fig 9 shows the algorithm results which is the view of the database resulting from algorithm software based on the 10-minute and 1-minute interval results. Hasan Dagi station provided different data interval measurements with the same anemometer and same point, allowing us to have a certain idea of which time interval the algorithm works more efficiently. Although the 1 and 10-min results are in 66% and 85% agreement with each other, the results from the 1-min measurements show more agreement with the radar. Averaging based on the 10-min results resulted in a loss in the detection of short-duration MB events. Another WS control method is shown in Fig 10. These methods are also applied to detect MB events for check.

### Wind measurement station results

**Fig 11:**
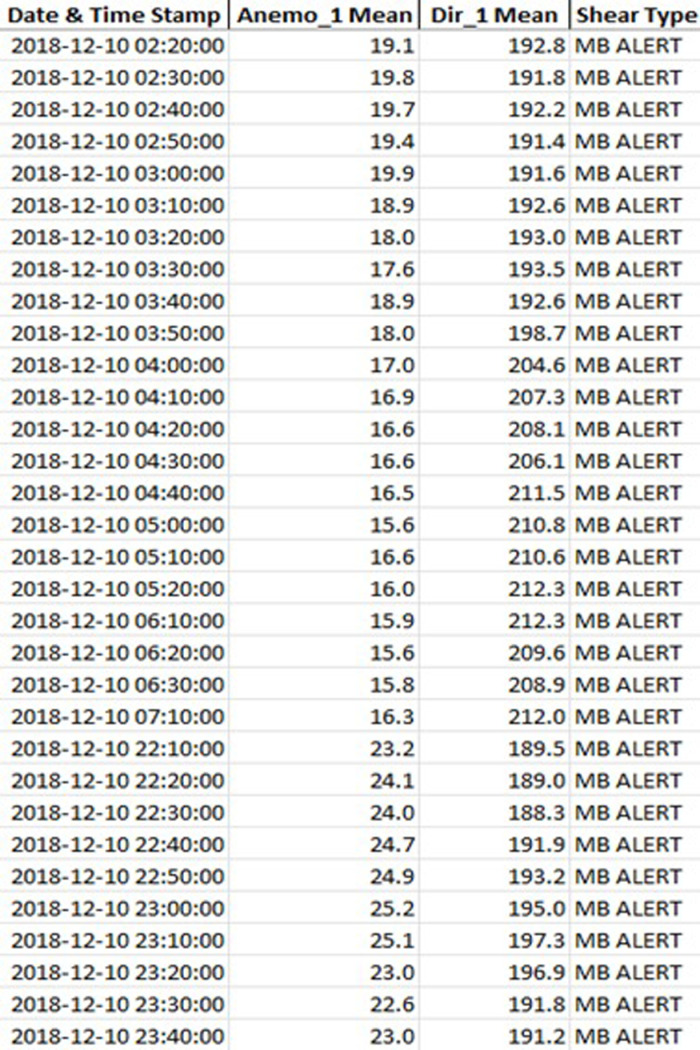
Microburst detection results of Wind Measurement Station. The table shows that part of matched wind shear events of the result Wind Measurement Station and Radar investigations.

The same algorithm has been applied also to data collected by wind measurement stations. 169 data lines belong to the MB event inside of 41407 data lines. It includes wind speed, wind direction, temperature, humidity, and pressure sensors. All data collected by these sensors have been analysed according to the wind shear detection. The data logger collected data between 08.02.2018 and 25.12.2018. 16 days of all measurement periods include MB events, and 23 MB events have been seen. Fig 11 shows part of the detected MB events by the algorithm. It should be noted that this part of the study has been handled in 10-minute intervals and almost 1-year period. All these measurement periods have covered 10-minute interval synoptic measurement. 9 days of MB events at the same time on the same day. These results show that a match with 56% was detected. In addition to the matching days as in the other results, temporally matching MB events within the days were compared independently. In this respect, the accuracy values were as successful as 70%. The remaining difference of approximately 30% is due to the 23 km distance between the stations.

### LIDAR measurement results

**Fig 12:**
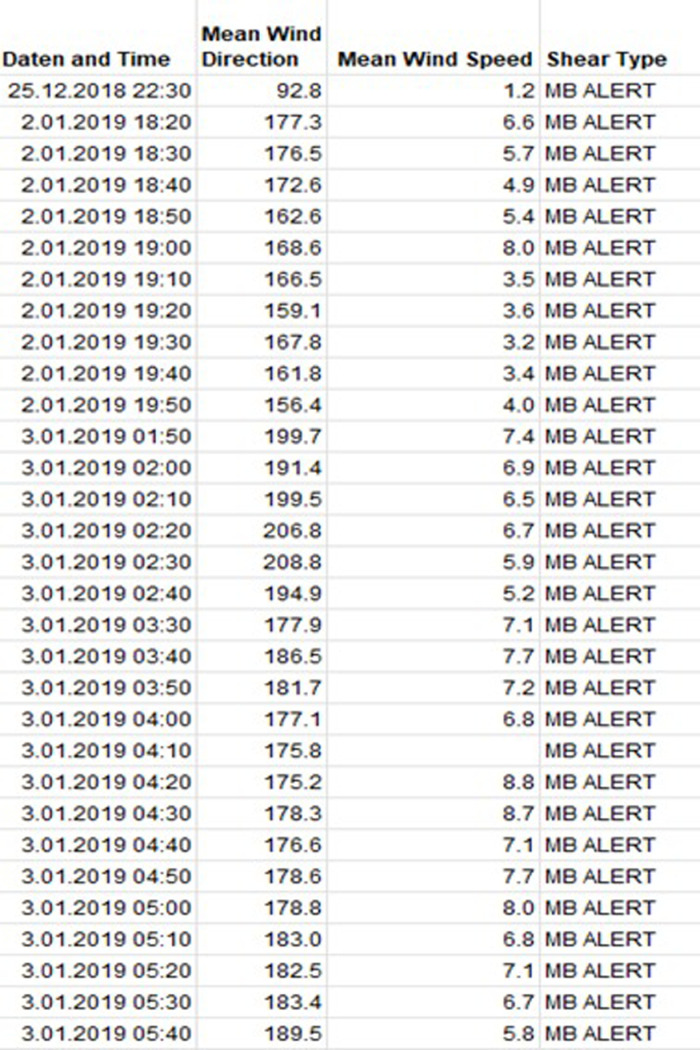
Microburst detection results of LIDAR. The table shows that part of matched wind shear events of the result LIDAR and Radar investigations.

The LIDAR data have collected the data between 08/02/2018 and 06/02/2019. 52325 data lines was processed by the algorithm. 1589 line has found MB event in 58 days. As a result of MB detection by LIDAR, 412 incidents were detected during the measurement period, which is higher than expected. Validation studies were conducted with a wind measurement station, 10 min synoptic station and 1 min synoptic station (Fig 12). A comparison was carried out by restricting the LIDAR data to the measurement dates of stations. When the reduced numbers are compared, the agreement rate is 5%, 75% and 41%, respectively. Although it is in the same location, the agreement rate with the wind measurement station is low.

### Radar MB detection results

**Fig 13:**
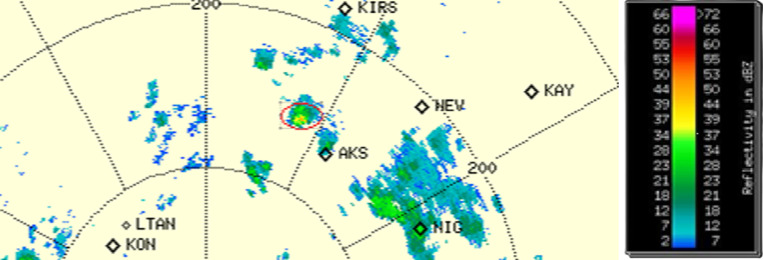
Karaman Weather Radar PPI Product.

Doppler weather radar products were evaluated for Karaman/Turkey (Fig 13). The colored areas on the map provide information about the air particles reflected backscatter to the radar antenna. The unit of reflection is dBZ. Groupings on the map indicate moving clouds. Hence, clouds with different speeds at short distance within the red circle in Fig 13. These are considered as MBs. Radar products was provided by the Turkish State Meteorological Service. 5 days’ data was processed according to the microburst detection aim. The red circle shows MB events close to the wind-measured area. Within the scope of the study, 1008 PPI products and reflection values and 3362 wind products were analysed during the MB radar analysis. 11 MB incidents were detected in 5 days. 2 different image signals were evaluated. The first one is that the reflection value of 55dBz and above fluctuates to small values due to the MB characteristics. Secondly, the MB event with a high probability of occurrence with the onset of precipitation was evaluated. The evaluation areas on the map are restricted as Synoptic and Wind Measurement points.

Fig 13. shows detected MB events time of point measurement data sets. It is clear from the graph that MB events differ over short distances. The left side shows the day in which WS was detected and the first hour-minute information of the day. Colors mark the distribution of WS detected with different devices. This graph shows the algorithm’s response to data at the same measurement locations. In addition, it allowed us to compare the 1-min and 10-min measurement intervals. It also allowed us to evaluate the performances of point measurement methods such as LIDAR and anemometer, which work on different principles, in the detection of MB events.

### Verification results

To evaluate the accuracy of the proposed method, statistical comparisons were made with other studies. The radar and LIDAR WS detection performance was investigated in particular. In addition, it should be noted that detailed events occurred at different sites from Aksaray/Turkey. Therefore, the verification study must be carried out with statistically different LIDAR and radar devices and specifically for the area where the devices are installed. Huang et al. have investigated LIDAR wind shear detection performance with Linear SVM, LDA, Logistic Regression and kNN methods with different parameters. The analysed data was collected in March 2018 by LIDAR at Hong Kong International Airport. Wind shear detection performance is between 49% and 91% with different parameters as mentioned methods [23]. Another experimental study has been conducted by Yuan et al. The data was collected by micro-pulse coherent Doppler wind LIDAR at Beijing Daxing International Airport. The data is from 1 April to 1 September 2021. Also, 9 anemometers have been located near the runway at the airport. These are also close to LIDAR. Before shear analysis, the authors conducted a correlation studies. The correlation coefficient is >  0.95 between the anemometer and LIDAR. As a result, when the detected low-level wind shear events were compared with the pilot reports, the agreement value was calculated as 100%. The detected event’s value is 10 for 66 days [43].

When looking at radar studies, the detected MB events differ in high accuracy rates and numbers. According to the 31-day radar performance as a similar period, 359 of 400 events could be detected. The probability of detection rate was 90% [44]. Tse Mei et al. presented another wind shear detection study using terminal Doppler weather radar. Furthermore, this study had a 91% probability of detection rate. The study covers the 24 hours on October 4^th^, 2015. 116 of 127 MB events were detected [45].

LLWAS has some accuracy arguments. One of them is probability of detection (POD). Another validation study is a statistical POD comparison. Detected MB sets from LIDAR, 10-min and 1-min analysed. Table 3 shows the POD values of the presented study including Jan’s results. Table 4 shows the POD values of all results.

**Table 3:** POD calculations of Jan.

POD (%)	LIDAR	1-min	10-min
LIDAR		100	80
1-min	12		10
10-min	100	100	

**Table 4:** POD calculations of all periods.

POD (%)	LIDAR	1-min	10-min
LIDAR		100	38
1-min	39		15
10-min	100	100	

## Conclusion

**Fig 14:**
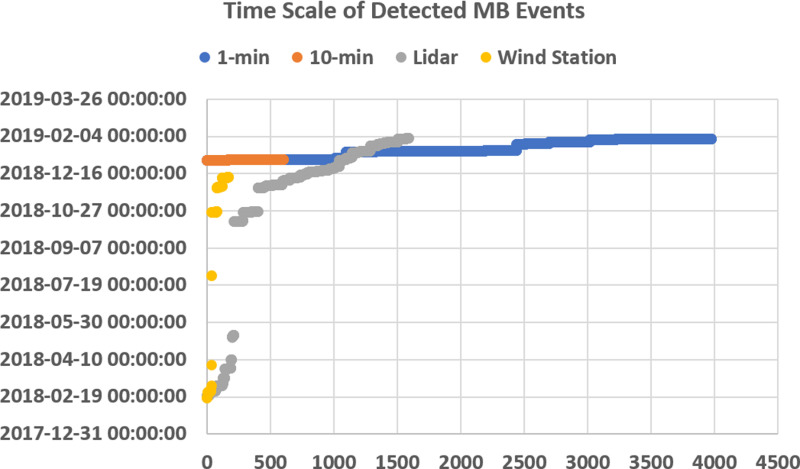
Time scale graph of detected MB events.

This paper provided a new algorithm for Microburst detection based on the point measurements method. The algorithm was tested using data from 3 different point measurement stations, two at the same point and the third 23 km away. The detected MB events showed good agreement in terms of number, days, hours and minutes. 5 days of radar data were analysed to validate the parameters in the algorithm. Using weather radar, the MB was detected, and the steps of the characterization process were checked. At the same time, the algorithm was compared by checking the number and duration of MB events detected at certain time intervals. It should be noted that this study provided us with various outcomes via big data analysis. In addition to the findings of the study, limitations and potential improvements are listed below,

- Developed new MB detection performance is promising for future studies. Firstly, MB events by anemometers matched with LIDAR results. This situation demonstrated the applicability of the algorithm with a single-point measurement data set in future studies.
- Repeating the presented algorithm with different data sets in different fields will contribute to the improvement of the algorithm.
- Hasan Dagi’s data was analysed via a developed algorithm. This station not only provided us with 2 more data sets to validate our overall analysis but also showed that the 1-minute data set gave more accurate results within the algorithm. The algorithm will be modified to run only for 1-minute data.
- The algorithm well matched the same point measurement as shown in Fig 14. 1-min and 10-min algorithms result in completely similar times which detected data lines. Likewise, the results of the LIDAR and wind station detected MB events time match each other at an acceptable level.
- However, there are differences in the frequency and dates of MB events. This proves that the MB event can vary greatly over a short distance. Also 5 days radar product limited the accuracy of point measurements MB detection calculations.
- In future studies, verification studies are planned to be carried out with LLWAS systems with 3D LIDAR and RADAR to increase the accuracy of the algorithm by using 1-minute values and single-point measurement points. However, the described areas are not common around the world.
- It is expected that the algorithm will be a support for LLWAS systems with tests to be carried out in different fields. In addition, it will provide detailed WS analysis for wind turbines in a way that has not been done before in the literature.

## Supporting information

S1 File Graph and result.
(XLSX)

S2 File Sub results.
(XLSX)

S3 File MB results monitoring.
(XLSX)

S4 File Minimal data det.
(XLSX)
